# Orientation-Dependent Deformation Behavior of 316L Steel Manufactured by Laser Metal Deposition and Casting under Local Scratch and Indentation Load

**DOI:** 10.3390/ma13071765

**Published:** 2020-04-09

**Authors:** Fabian Pöhl, Corinna Hardes, Felicitas Scholz, Jan Frenzel

**Affiliations:** 1 Chair of Materials Technology, Faculty of Mechanical Engineering, Ruhr-Universität Bochum, 44801 Bochum, Germany; hardes@wtech.rub.de; 2 Chair of Materials Science and Engineering, Faculty of Mechanical Engineering, Ruhr-Universität Bochum, 44801 Bochum, Germany; felicitas.scholz@rub.de (F.S.); jan.a.frenzel@rub.de (J.F.)

**Keywords:** laser metal deposition, 316L, scratch testing, nanoindentation, grain orientation

## Abstract

This study analyzes the local deformation behavior of austenitic stainless steel 316L, manufactured conventionally by casting and additively by laser metal deposition (LMD). We produced directionally solidified 316L specimens with most grains showing (001) orientations parallel to the longitudinal specimen axis. We conducted nanoindentation and scratch experiments for local mechanical characterization and topography measurements (atomic force microscopy and confocal laser scanning microscopy) of indentation imprints and residual scratch grooves for the analysis of the deformation behavior and, in particular, of the pile-up behavior. The local mechanical properties and deformation behavior were correlated to the local microstructure investigated by scanning electron microscopy with energy dispersive X-ray spectroscopy and electron backscatter diffraction analysis. The results show that the local mechanical properties, deformation behavior, and scratch resistance strongly depend on the crystallographic orientation. Nearly (001)-oriented grains parallel to the surface show the lowest hardness, followed by an increasing hardness of nearly (101)- and (111)-oriented grains. Consequently, scratch depth is the greatest for nearly (001)-oriented grains followed by (101) and (111) orientations. This tendency is seen independently of the analyzed manufacturing route, namely Bridgman solidification and laser metal deposition. In general, the laser metal deposition process leads to a higher strength and hardness, which is mainly attributed to a higher dislocation density. Under the investigated loading conditions, the cellular segregation substructure is not found to significantly and directly change the local deformation behavior during indentation and scratch testing.

## 1. Introduction

Due to the possibility of creating near-net shape components on demand, laser-based layer-by-layer densification of metallic powders by means of additive manufacturing is being increasingly used in a broad range of industrial applications. Among a variety of commercially available devices, direct energy deposition techniques such as laser metal deposition (LMD), and laser powder bed fusion processes such as selective laser melting (SLM), have become one of the most attention-gaining additive manufacturing routes over the last few years [1]. Whereas powder bed fusion techniques enable the fabrication of complex and filigree geometries, LMD is used to build up larger components and is furthermore beneficial in terms of repair [2]. Due to the need for pre-alloyed, gas-atomized, and screened powder particles, additive manufacturing is a cost-intensive process with respect to high facility and equipment expenses [2], and can mostly be afforded by sectors with large investment capacities such as aerospace [3], medicine [4], or tooling [5]. 

During laser metal deposition, a metal powder is fed by a nozzle and an argon gas jet into the local laser weld bath on the component to be produced. This process enables local buildup of a component by the systematic movement of the laser. In contrast to microstructures processed by conventional casting, the process is characterized by the melting of powder particles with a subsequent high cooling rate. Furthermore, there is a process-dependent repetitive remelting and reheating of already solidified structures with overlapping heat-affected zones. This leads to unique microstructures solidified away from thermodynamic equilibrium, with high residual stresses that are highly dependent on the processing parameters and the building direction. Austenitic stainless steel 316L is frequently used to analyze the process, the resulting microstructures, and properties as well as the production of entire components in use [6]. After LMD processing, the microstructure of 316L is hierarchical. Depending on the processing parameters, the microstructure consists of equiaxed grains and stalk-shaped grains epitaxially grown towards the temperature gradient on a mesoscale, a subcell structure caused by segregations on a micro- and nanoscale, as well as oxide inclusions with sizes on the submicron and nanometer length scale [7]. The polycrystalline microstructure can have a significant texture that depends on the processing parameters. At the same time, LMD microstructures have a higher dislocation density compared to more slowly solidified microstructures during conventional casting. As a result, these microstructures have a higher hardness and strength than conventional microstructures [8]. However, defects such as pores and cracks in additively-manufactured microstructures have a major impact on the fatigue behavior under cyclic loading conditions [9,10]. In past years, the mechanical behavior of additively-manufactured 316L steel was extensively analyzed by materials testing under several loading conditions, such as hardness testing, tensile testing, compression testing, impact testing, or compact tension testing [11,12,13,14]. However, the tribological behavior of additively-manufactured steel has been investigated significantly less frequently. Examples of the tribological behavior of additively-manufactured 316L are limited to cavitation erosion behavior [15], and mostly to sliding wear [16,17,18]. Although 316L steel is not a typical choice for an abrasion-resistant material, abrasive wear may play a role in certain applications. Furthermore, complex microstructures with higher strength, higher hardness, and high strain-hardening potential could even qualify austenitic 316L for applications under mild abrasive wear conditions. Practically nothing is known about the abrasive wear resistance and the deformation behavior under local scratch load of 316L specimens or components entirely manufactured by LMD.

Under abrasive loading conditions, conventionally cast austenitic microstructures show pronounced plastic deformation, with the microploughing and microcutting micromechanisms being active. Furthermore, the crystallographic orientation was found to exert a strong influence on local mechanical and tribological behaviors [15]. Indentation in grains close to (111) orientation reveal that this orientation has the highest hardness, followed by (101) and (001) orientations with decreasing hardness values [19]. These local differences in mechanical behavior can greatly influence tribological behavior. Indentation testing can also be used to study local deformation behavior by analyzing the pile-up behavior next to indentation imprints. Austenitic steels show a low pile-up tendency, due to their high strain-hardening potential. However, on the micro- and nanoscale, there is also a strong influence of the crystallographic grain orientation on the deformation and pile-up behavior based on crystal plasticity theory [20,21]. The tribological and local mechanical properties are well characterized for conventionally manufactured 316L. For hierarchical microstructures after LMD, the effect of crystallographic orientation, of cellular segregation substructure, or higher dislocation density on local mechanical behavior, particularly under a scratch load, is not known.

In this study, we have focused on local mechanical behavior during scratch and indentation testing of additively-manufactured (LMD) 316L microstructures, in comparison to conventionally cast, hot-rolled, and solution-annealed, as well as directionally solidified, 316L microstructures. After processing, the microstructures were first analyzed by scanning electron microscopy (SEM) with electron backscatter diffraction analysis (EBSD). For selected grain orientations in polycrystalline microstructures after LMD and conventional casting, scratch depth, pile-up behavior next to the scratch groove (measured by atomic force microscopy and confocal laser scanning microscopy), active abrasion micromechanisms, and indentation hardness were analyzed and compared. In the directionally solidified microstructures, there are only nearly (001)-oriented grains parallel to the longitudinal specimen axis. These grains were also analyzed with respect to the aforementioned micromechanical behavior and parameters, as well as a variation in scratch angle on the specimen surface. We also analyzed the indentation size effect (ISE). Its strong occurrence in austenitic steels is well known [22]. However, its characterization after the LMD process is lacking. For this purpose, the hardness was analyzed as a function of indentation depth, measured by continuous stiffness measurement (CSM), to quantify the extent of the indentation size effect in the different microstructures.

## 2. Materials and Methods

### 2.1. Material and Processing

**Figure 1:**
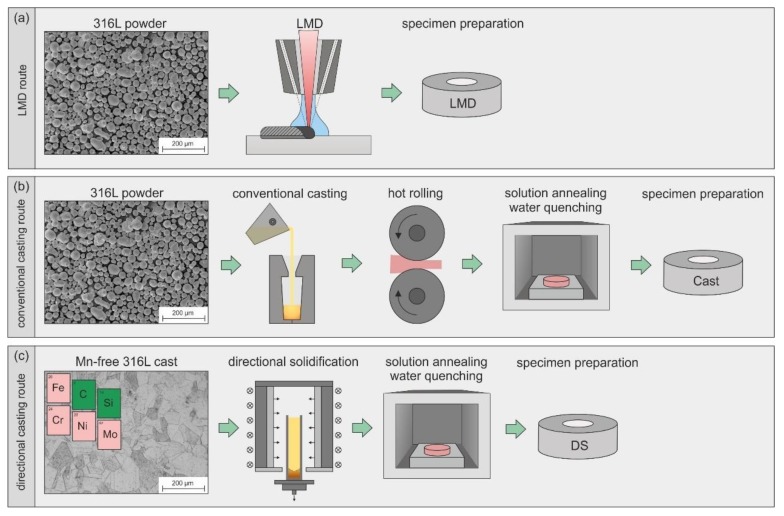
Schematic illustration of the (**a**) laser metal deposition (LMD) processing route, (**b**) conventional casting route, and (**c**) directional casting route.

We investigated austenitic stainless steel 316L that had been subjected to three different processing routes. As Figure 1 illustrates, the main difference between the routes is primary shaping, which was conducted by LMD (a), conventional casting (b), and directional solidification (c). According to the processing routes, specimen nomenclature is thus LMD (a), Cast (b) and DS (c).

LMD processing was conducted on a three-axis LMD setup with a fiber laser (wavelength 1070 nm), a focus of 1 mm, and a power of 315 W (Table 1). 

**Figure 2:**
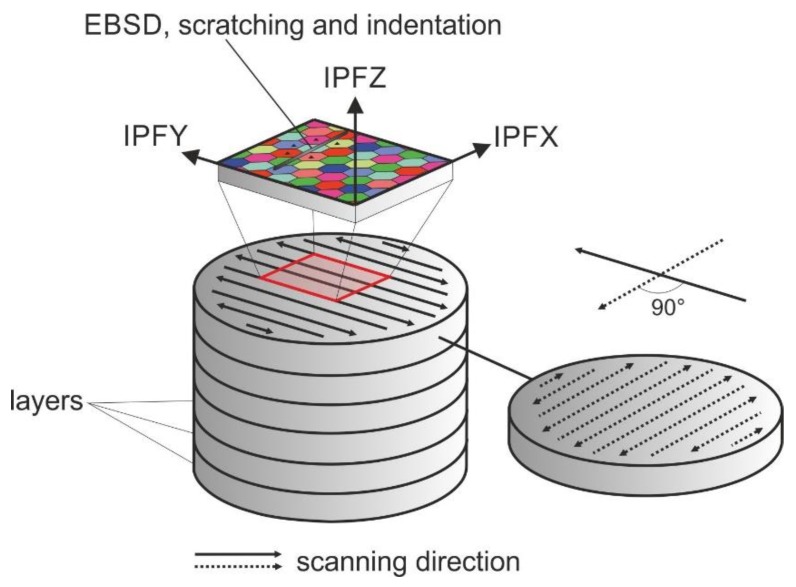
Schematic of geometric relationships of the specimens built by LMD.

The powder feed rate into the melt pool was 2.3 g/min, with a feed rate for the process head of 10 mm/s. The flow rate of shielding gas (Ar) and carrier gas were 10 L/min and 3 L/min, respectively. As indicated in Figure 2, hatching was 0.5 mm with a 50% overlap, a layer height of 0.3 mm, a zigzag strategy, and a changing welding direction of 90° for each layer. 

The chemical composition of the LMD specimens is given in Table 2.

To retain and analyze the hierarchical microstructure, no further heat treatment was conducted. Specimens were directly embedded in conductive phenolic resin with graphite, ground with SiC paper, and polished with a diamond suspension to a final polishing step with an average diamond grain size of 1 µm. For micromechanical characterization and EBSD analysis, a further polishing step with colloid amorphous silicates, with an average grain size of 0.02 µm, was included.

As a reference, 316L was processed by conventional casting of a 200 g ingot. We used the same gas-atomized 316L powder as that processed by LMD to ensure an almost identical chemical composition (Table 2). For homogenization, the ingot was hot-rolled in eight rolling steps from an initial thickness of 32.5 mm to a final thickness of 15.5 mm (rolling temperature of 1150 °C). Between each rolling step the specimen was rotated by 90° to reduce texture development. Hot rolling was followed by solution annealing at 1050 °C for 30 min and water quenching to avoid carbide precipitations. Subsequent specimen preparation was identical to that of the LMD specimens.

Directional solidification processing was conducted in a Bridgman furnace (KZV-A40-400/161G-V) from Gero GmbH, Neuhausen, Germany. We used a nominal temperature of 1570 °C and vacuum of 3 × 10^−4^ mbar for melting. Directional solidification was conducted with a constant withdrawal rate of 180 mm/h. The temperature gradient was close to 13 K/min. To minimize the evaporation of Mn during Bridgman processing, the chemical composition was slightly adjusted, as given in Table 2. The process was followed by solution annealing with water quenching and specimen preparation equal to that of the LMD and Cast specimens. Further information on the Bridgman furnace is given in [23]. 

### 2.2. Nanoindentation

**Figure 3:**
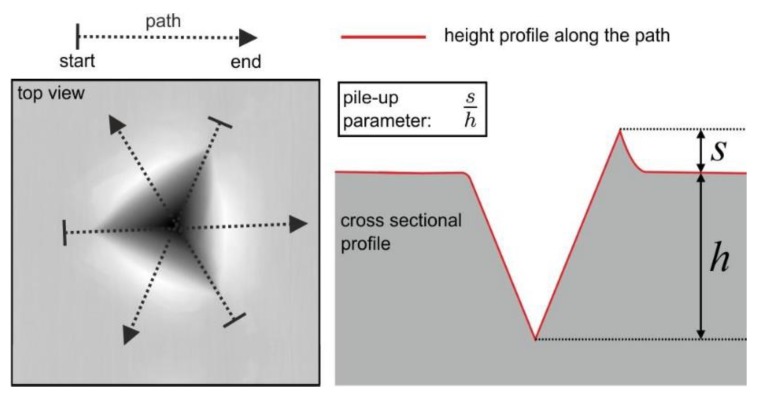
Schematic illustration of a residual indentation imprint and the three paths used to extract height profiles to determine the normalized pile-up parameter *s/h*. The average of the height profiles along the three illustrated paths was calculated for each indent.

Nanoindentation experiments were conducted with an iMicro nanoindenter from Nanomechanics Inc. (Oak Ridge, TN, USA) equipped with a diamond Berkovich tip. The loading and unloading rates were constant with 0.2 s^−1^, and the maximum load was 20 mN. Measured load-displacement curves (P-h curves) were evaluated according to the Oliver and Pharr method to determine the hardness *Hi* [24,25]. The SEM-EBSD technique was used for pinpointing the grains with suitable crystallographic orientation for subsequent nanoindentation testing. In addition to the hardness, the loading curvature *C* was calculated from the *P*-*h* curves. The loading curvature is a constant parameter (given material and self-similar indenter) of the relationship between load and depth of the loading curve [26,27]. This parameter is connected to elastic-plastic material behavior, such as Young’s modulus, yield stress, and strain hardening behavior in a complex manner. Another evaluated parameter was the normalized pile-up height *s*/*h* (Figure 3).

To calculate *s*/*h*, the topography images of residual indentation imprints were measured by atomic force microscopy and evaluated as show in Figure 3. For each indent, the pile-up parameter was calculated from the average of three individual height profiles, as illustrated in Figure 3. To analyze the indentation size effect (ISE), the CSM method (continuous stiffness measurement) was used to measure hardness as a function of the indentation depth [28].

### 2.3. Scratch Testing

**Figure 4:**
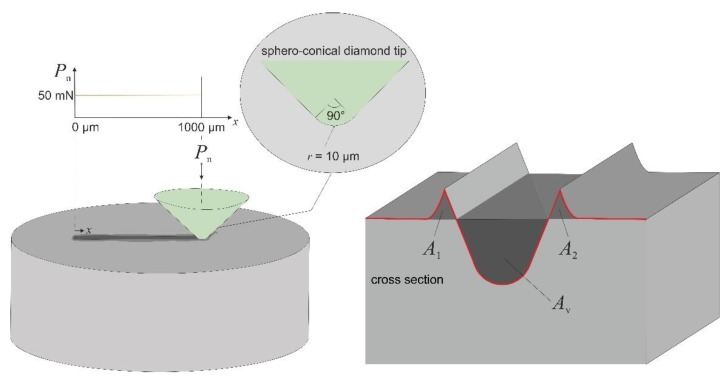
Schematic illustration of the spheroconical diamond indenter scratching the material surface with a constant normal load of 50 mN. The *f*_ab_ parameter is calculated by the area of the scratch groove furrow *A*_v_ and the areas *A*_1_ and *A*_2_ caused by piled-up material, Equation (1).

Scratch tests were conducted with a scratch tester (CSM instruments; NST module, Peseux, Switzerland) equipped with a spheroconical diamond (tip radius 10 µm, Figure 4). We used a constant normal load of 50 mN and a scratch length of 1 mm. Scratch speed was constant at 300 µm/min. Suitable analysis positions for topography measurements were chosen along the scratch groove on the basis of SEM analysis with EBSD measurements. Topography measurements were conducted by atomic force microscopy and confocal laser scanning microscopy. Based on these measurements, the residual scratch depth, pile-up behavior, and the abrasion *f*_ab_ parameter after Zum Gahr were characterized [29]. The *f*_ab_ parameter was determined by analyzing cross-sectional height profiles, as illustrated in Figure 4. The *f*_ab_ parameter is given by Equation (1) and relates the areas of the pile-up (*A*_1_ and *A*_2_) to the area of the scratch groove furrow (*A*_v_) for a given cross-section of the scratch groove (Figure 4).

(1) $$f_{\mathrm{ab}}=\frac{A_{v}-\left(A_{1}+A_{2}\right)}{A_{v}}$$

### 2.4. Atomic Force Microscopy and Confocal Laser Scanning Microscopy

The topography and pile-up behavior of the indentation imprints and the scratch groves were characterized by an atomic force microscope (AFM) from Bruker (Santa Barbara, CA, USA), in the contact mode (type Nanos) and a confocal laser scanning microscope (CLSM) from Keyence, Osaka, Japan (type VK-X 200). AFM images were post-processed with Gwyddion software (version 2.47) and CLSM images with MultiFileAnalyser software from Keyence, Osaka, Japan (version 1.3.1.120).

### 2.5. Scanning Electron Microscopy

A scanning electron microscope (SEM, Mira 3, Tescan, Brno, Czechia) operating at 15 kV and additionally equipped with energy dispersive X-ray spectrometer (EDS, OXFORD, X-Max 50, Abingdon, UK) and electron back-scatter diffraction (EBSD, Oxford, Nordlysnano, Abingdon, UK) were used to characterize the microstructure of the investigated materials, the scratch grooves, and indentation imprints, and to analyze the crystallographic orientation. For EBSD analysis, we used an acceleration voltage of 15 kV, a working distance of 15 mm, and a step size of 0.2 μm.

## 3. Results and Discussion

### 3.1. Microstructure

**Figure 5:**
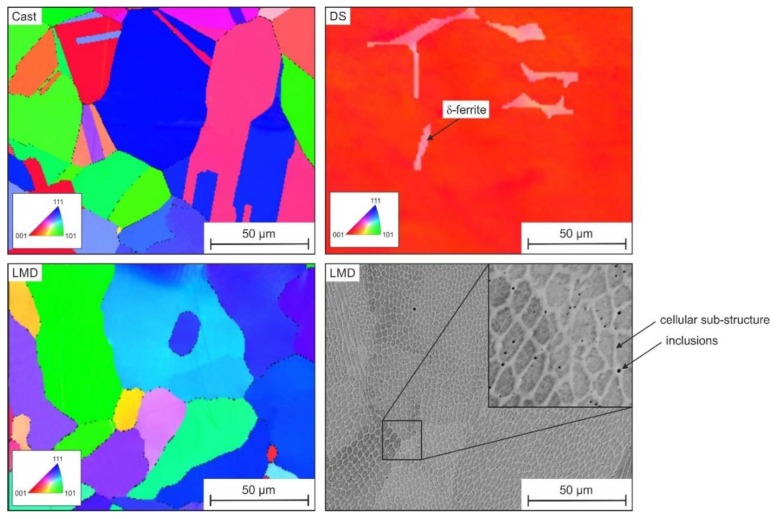
Electron backscatter diffraction analysis (EBSD) images (ND-IPF, parallel to the longitudinal axis of the directional solidification (DS) specimen) of the investigated microstructures. The cellular substructures and inclusions after LMD processing are shown in backscattering electron contrast (BSE).

Figure 5 shows the microstructure of the investigated specimens. After conventional casting, hot forming, and solution annealing, the microstructure consisted of homogeneous austenite, with randomly distributed and relatively coarse globular grains and twins.

**Figure 6:**
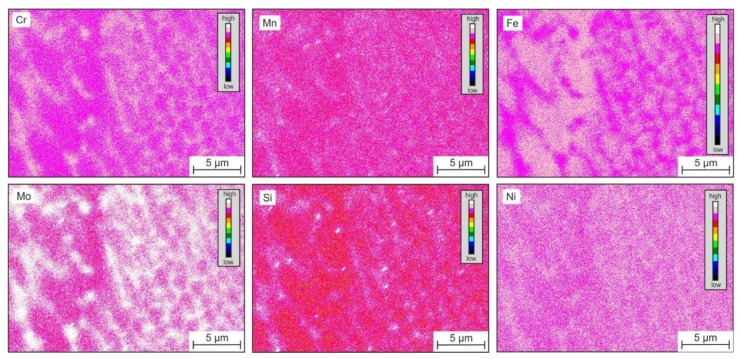
Qualitative energy dispersive X-ray spectrometer (EDS) element mapping shows the cellular segregation substructure developed during LMD processing.

**Figure 7:**
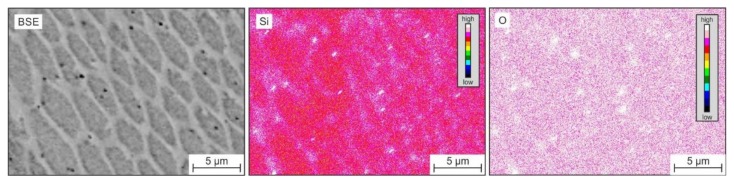
Qualitative EDS element mapping shows silicon-rich oxide inclusions in the LMD specimen. The BSE image corresponds to another position in the microstructure.

The microstructure after directional solidification and solution annealing had long and directional grains that were nearly (001)-oriented parallel to the surface. However, the microstructure contained small amounts of delta ferrite, resulting from slow cooling during casting. Since micromechanical testing was only conducted in the austenite phase, the delta ferrite had no effect on the results. The LMD process with local melting, rapid cooling, and repetitive heating during processing of the individual layers led to a polycrystalline anisotropic austenitic microstructure, with columnar grains having a unique segregation subcell structure within the individual grains. On the nanometer scale, there were oxide inclusions (see black spherical phases in Figure 5). The EDS mapping in Figure 6 reveals that the cellular substructure was caused by segregations, whereas elements such as Cr, Mo, Mn, and Si were enriched at the cell walls. Based on the EDS results given in Figure 7, the nanometer-scale inclusions could be considered silicon-rich oxides [7,8,14].

### 3.2. Scratching and Indentation

**Figure 8:**
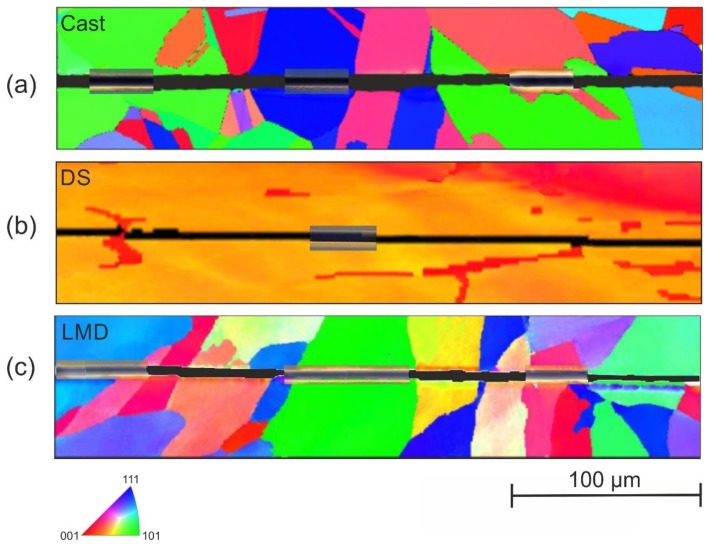
EBSD images of the investigated microstructures after scratching with a spheroconical diamond tip (*r* = 10 µm) and a normal load of 50 mN with qualitative superimposition of atomic force microscope (AFM) topography images at different positions along the scratch groove.

Figure 8 depicts the residual scratch groove in the polycrystalline microstructure of the specimens Cast (a), DS (b) and LMD (c). 

**Figure 9:**
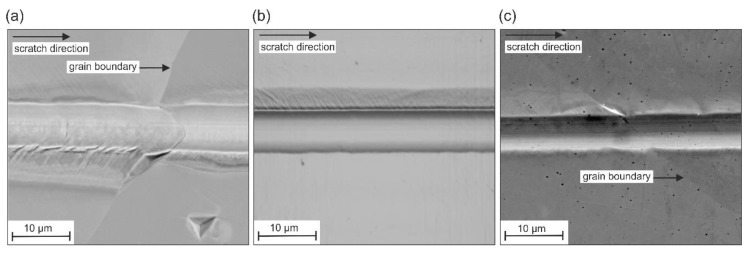
SEM images of residual scratch grooves in (**a**) Cast, (**b**) DS, and (**c**) LMD microstructures.

As revealed by the superimposed AFM images and additionally visible in Figure 9a,c, the deformation behavior was influenced by the crystallographic orientation. The deformation behavior of the specimen SD is homogeneous along the scratch distance (Figure 9b).

Within individual grains, the scratch depth, width, and pile-up formation was relatively constant. However, scratching along a grain boundary led to a change in the deformation processes. This was mostly visible through a changed pile-up formation next to the scratch groove.

**Figure 10:**
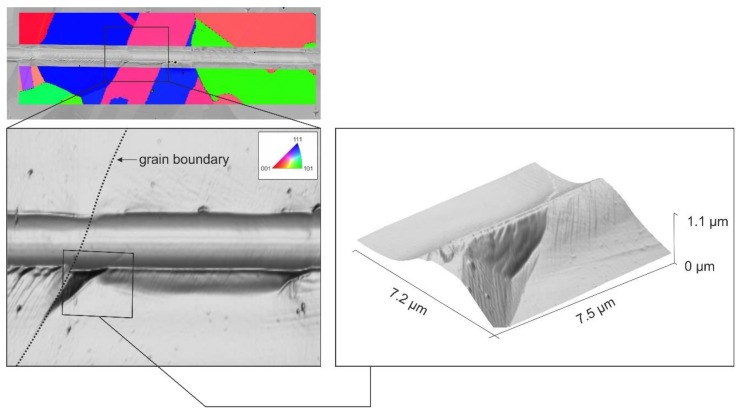
Localized pile-up formation at a grain boundary caused by scratching the polycrystalline microstructure of the LMD specimen (EBSD/BSE image and AFM 3D topography images).

An accumulation of plastic deformation with a distinct pile-up formation was often seen at a grain boundary. One example of such a behavior is displayed in Figure 10. 

Plastic deformation was strongly dependent on the given slip systems, and hence, on their orientation with respect to the superimposed loading. Based on Schmidt’s Law, scratch loading leads to different resolved shear stresses in the glide systems. The values of the Schmidt factors vary as a function of crystallographic orientation, which leads to orientation-dependent anisotropic plastic behavior. Due to incompatible glide systems at the grain boundary, the material was piled-up rather than continuously flowing forward in the neighboring grain.

**Figure 11:**
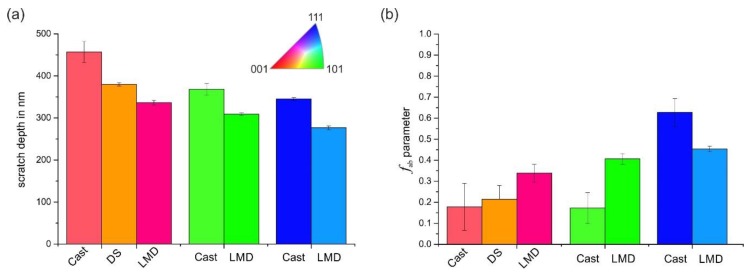
(**a**) Scratch depth and (**b**) *f*_ab_ parameter of the investigated specimens and grain orientations.

The differing mechanical behavior of the individual grains was also visible in the residual scratch depth. We used AFM measurements to determine the scratch depth of individual grains of the cast and LMD specimen. The result is given in Figure 11a, from which the following observations could be made. Firstly, the scratch depth depended on the crystallographic orientation, and it generally decreased from grains closely (001)-oriented over (101)- to (111)-oriented grains with the smallest scratch depth. Secondly, this tendency was observed for both processing routes (Cast and LMD). Thirdly, the scratch depths of the LMD specimen were significantly smaller than those of the cast specimens. This result indicated a beneficial effect of the LMD processing route on scratch resistance. As a first tendency, a reduced scratch depth led to a reduction in mass loss during abrasion.

**Figure 12:**
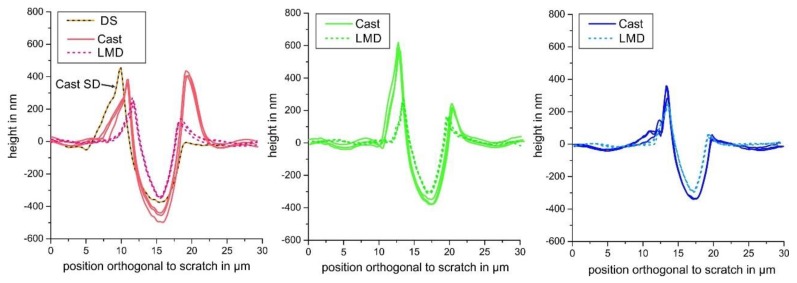
Height profiles orthogonal to the scratch groove in the different specimens and grain orientations.

However, not only the scratch depth was considered to characterize the scratch resistance. According to Zum Gahr, not all of the displaced material volume of a scratch groove is directly chipped out during scratching [29]. For sufficiently ductile metallic materials, greater volumes are ploughed to the sides forming pile-up. The *f*_ab_ parameter gives an estimate of the amount of pile-up. As Figure 11b illustrates, the *f*_ab_ parameter showed a roughly increasing tendency in the order (001)- (101)-(111). However, the scatter was high and there was no clear correlation. Generally, a small *f*_ab_ parameter indicates large plastic deformation with distinct pile-up formation with small volumes of chipped out material. For the investigated specimens, all determined *f*_ab_ parameters were relatively small, especially for the closely (001)- and (101)-oriented grains. A clear difference between Cast and LMD specimens could not be identified. Since the *f*_ab_ parameter was comparable between Cast and LMD, it was concluded that the resistance to scratching — as an ideal abrasion process of the LMD microstructure — was higher due to the reduced scratch depth. The similar pile-up behavior was also revealed by height profiles orthogonal to the scratch, as illustrated in Figure 12. 

**Figure 13:**
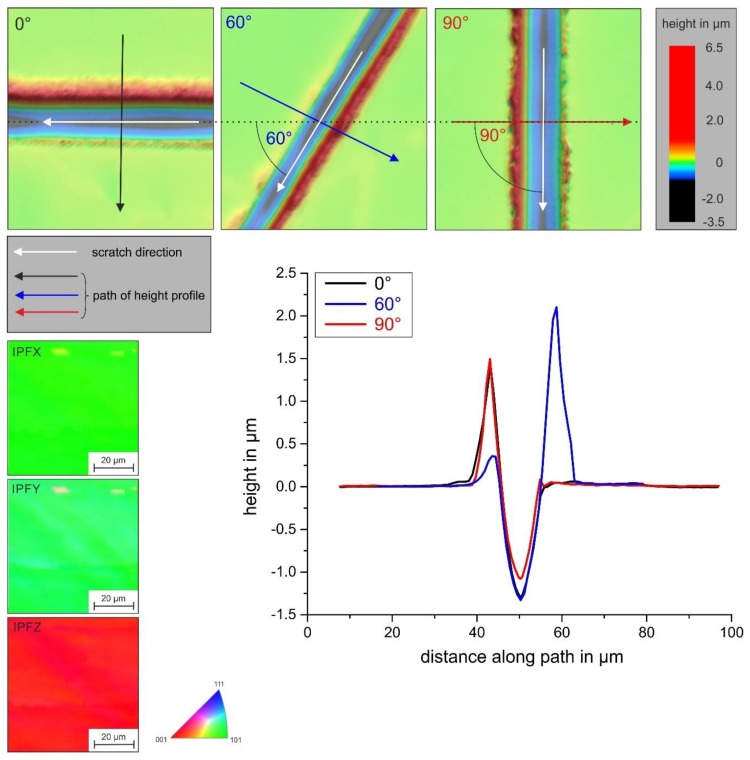
Pile-up formation in the nearly (001)-oriented DS specimen (parallel to the longitudinal specimen axis) as a function of scratch orientation.

This figure also shows the reduction in the scratch depth in the order (001)-(101)-(111). However, it is important to mention that the pile-up tendency, and thus the *f*_ab_ parameter, was also dependent on the scratch direction within an individual grain with a given orientation. As Figure 13 proves, the pile-up behavior varied in the nearly (001)-oriented grain of the DS specimen as a function of the scratch direction. 

The strong tendency of the (001) orientation for significant pile-up formation remained unchanged, whereas the degree of pile-up at each side next to the scratch groove varied. This demonstrated once more that the plastic deformation was controlled by activation of the given glide system of the crystal, and hence a superimposition of load and crystallographic orientation.

**Figure 14:**
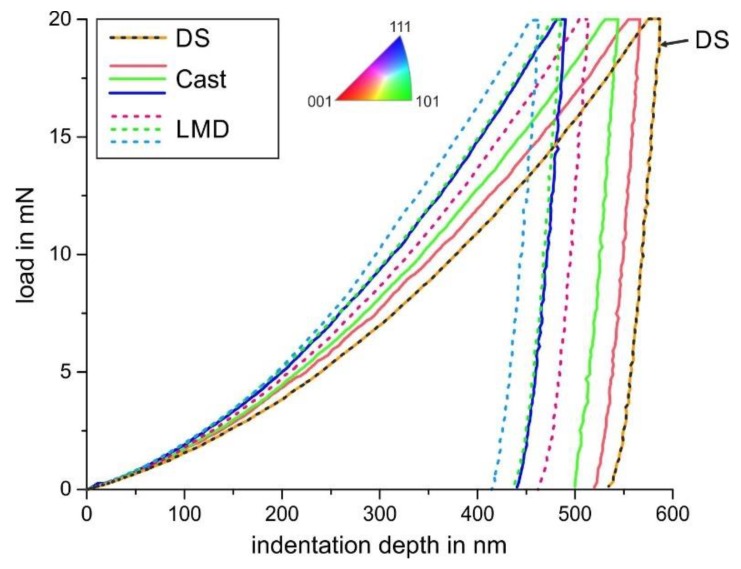
Load-displacement curves of the investigated specimens and crystallographic orientations.

**Figure 15:**
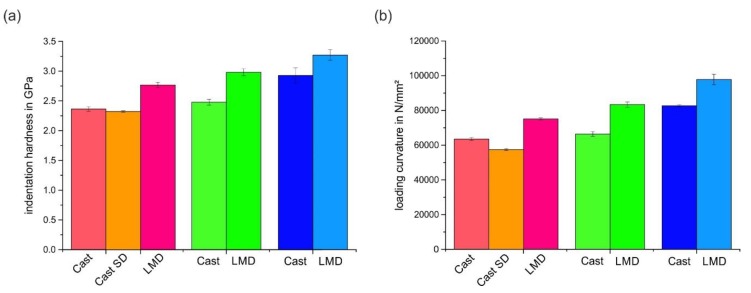
(**a**) Hardness *H_i_* and (**b**) loading curvature *C* of the investigated specimens and crystallographic orientations.

There was also a correlation of the local scratch deformation and scratch depth to the micromechanical properties measured by nanoindentation. Figure 14 and Figure 15 show the mean load-displacement curves and calculated hardness, as well as loading curvature of the investigated grains for the Cast and LMD specimens. 

**Figure 16:**
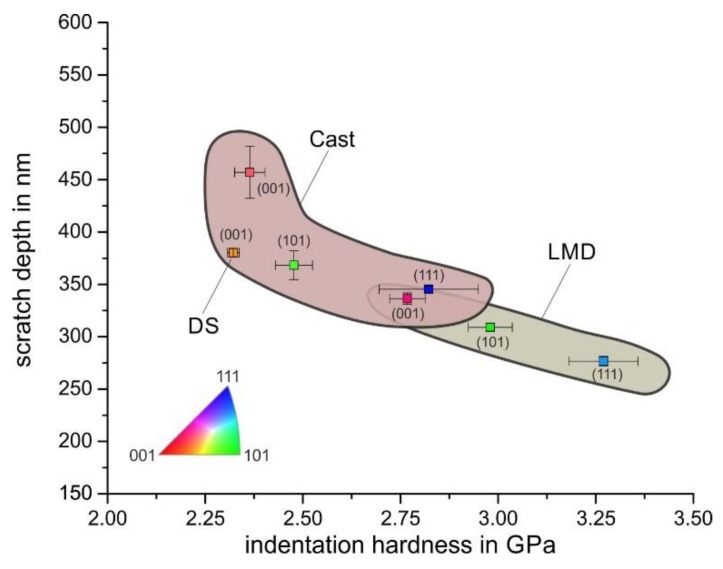
Relationship between scratch depth and hardness for different crystallographic orientations and processing routes.

The following observations could be made from these results. Firstly, for a given load, the local strength is a function of the crystallographic orientation, and grains with close (001) orientations have the lowest hardness and *C* parameter, followed by (101) and (111). This is in agreement with the experimental results of Chen et al. on an austenitic model alloy [19]. Secondly, for an equal crystallographic orientation, the LMD process led to a higher strength and hardness as well as C parameter. Grains with higher hardness showed a smaller scratch depth, because their resistance to penetration was higher. Hence, there was an inhomogeneous local abrasive behavior on the microscale (scratch width smaller than the grain size) within the individual polycrystalline microstructures. The higher strength of the LMD specimen led to a reduction in the scratch depth and not to a significant change in the active micromechanisms. Thus, the LMD microstructure was considered to have a higher scratch resistance and, under similar abrasive loading, a higher abrasive wear resistance. Figure 16 illustrates the relationship between hardness and scratch depth as a function of crystallographic orientation and specimen processing. 

The DS specimen might not directly be comparable because of the slightly changed alloy composition (reduced Mn content). Although the DS specimen had the lowest hardness, its scratch depth was not the highest. This might have resulted from a different mechanical behavior caused by the changed alloy composition.

As main reasons for the higher hardness and reduced scratch depth after LMD processing (in comparison to the cast specimen), a higher dislocation density and the cellular substructure were considered. Thome et al. found a high density of geometrically necessary dislocations at grain boundaries in a Ni-base superalloy after additive manufacturing using selective electron laser beam melting [30]. The oxide inclusions with sizes in the range of ~100 nm were not believed to have a significant impact on the strength of the LMD specimen. Thus, a possible higher dislocation density and the cellular substructure were considered more closely in the following.

**Figure 17:**
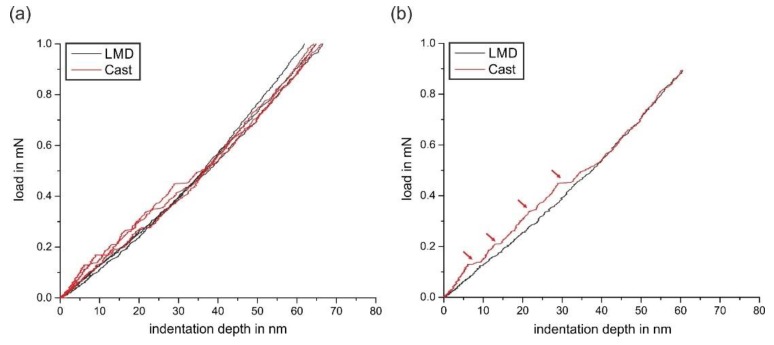
(**a**) Several load-displacement curves measured in the LMD and Cast specimens, and (**b**) one selected curve of both specimens for better comparison. At the beginning of the curve, pronounced pop-ins are visible in the case of the specimen cast.

A higher dislocation density after LMD processing in comparison to conventional casting is indirectly seen at the very beginning of the load-displacement curves. Figure 17 illustrates the occurrence of multiple pop-in phenomena in the loading curves of the cast specimen. 

In contrast, no pop-ins were found for the LMD specimens. At the beginning of the loading curves, pop-ins are often caused by dislocation nucleation and multiplication with a sudden displacement burst [31]. This is most likely to occur when the stress field during indentation includes a volume that is initially free of dislocation sources. Since pop-in phenomena are suppressed after LMD processing, the dislocation density after LMD processing might be higher.

**Figure 18:**
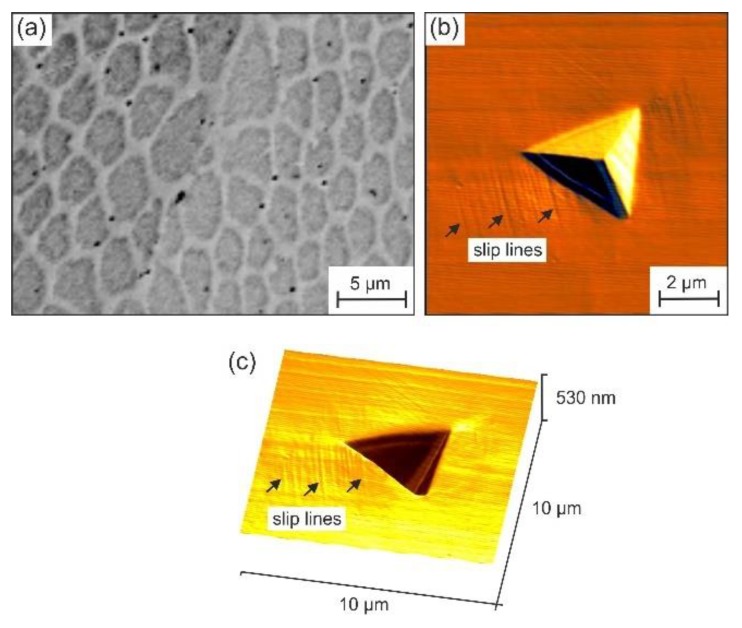
Images of the LMD specimen: (**a**) BSE image of the etched cellular substructure, (**b**) top-view AFM image of an indentation imprint with parallel slip lines in the plastic zone next to the imprint, and a (**c**) 3D visualization of the indentation imprint with parallel slip lines in the plastic zone next to the imprint.

The cellular substructure could also influence the local strength and deformation behavior. However, a direct effect of this was not seen on the local or global deformation behaviors. As Figure 18 shows, the slip lines on the surface next to indentation imprints were not visibly influenced in their appearance by the substructure. 

**Figure 19:**
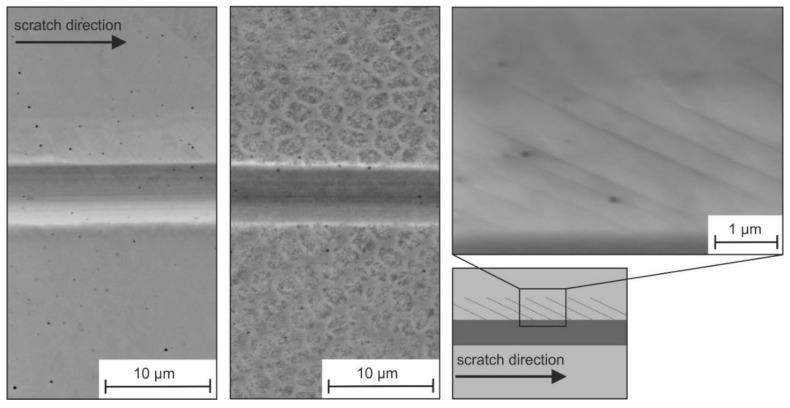
SEM images of a scratch groove in the LMD specimen.

**Figure 20:**
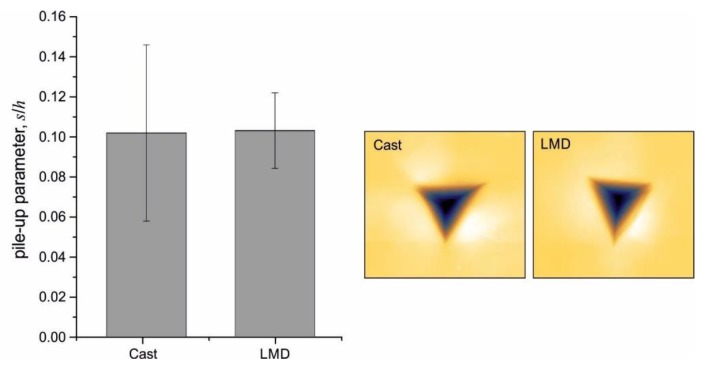
Normalized pile-up parameter and top-view AFM image of the Cast and LMD specimens.

This also holds true for the deformation next to the scratch grooves (Figure 19). A comparison of the normalized pile-up parameter also did not show a significant difference between the LMD and cast specimens (Figure 20).

These results indicated that the cellular substructure did not directly change the general deformation behavior under local indentation and scratch loading. Slip lines and plastic flow characteristics were not noticeably affected by the substructure. However, the substructure could have indirectly influenced the deformation behavior by locally influencing the dislocation density. TEM investigations showed an increased dislocation density of additively manufactured 316L at the cell walls [7].

**Figure 21:**
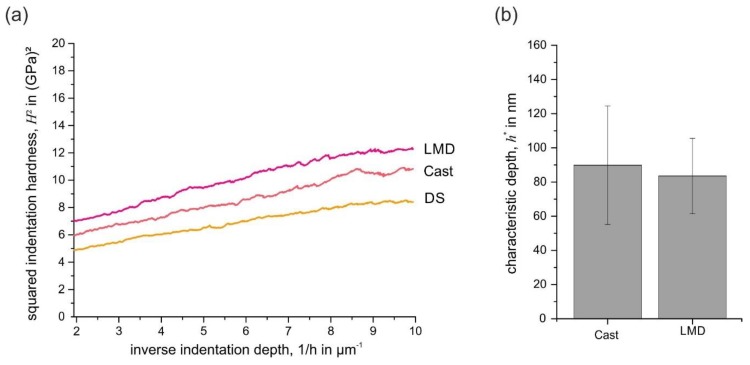
(**a**) Nix-Gao plot of nearly (001)-oriented grains and (**b**) calculated characteristic depth h* calculated from at least three indentation experiments (CSM method).

Finally, the indentation size effect (ISE) was investigated in nearly (001)-oriented grains. During plastic deformation on the micro- and nanoscale, size effects play an important role, especially during indentation of austenitic steels [22]. In order to investigate the influence of processing route on the ISE, nearly (001)-oriented grains of the LMD, Cast, and DS specimens were analyzed. Figure 21a shows a Nix-Gao plot of the closely (001)-oriented grains [32]. According to the model, the ISE was expressed by a linear relationship between the squared hardness and the inverse indentation depth. 

The linear relationship with positive slope in Figure 21a visualizes the presence of an ISE for all processing routes. Since the curves have a similar slope and are parallel with good agreement, the ISE for (001)-orientated grains appeared to be similar, independently of the processing route. This visual impression was quantitatively confirmed by the calculation of the characteristic length scale h* of the Nix-Gao model. The higher h* is, the stronger is the ISE. As Figure 21b proves, the parameter was very similar, independent of the processing route.

## 4. Summary and Conclusions

In this study, we investigated the local mechanical behavior during scratch and indentation testing of a additively-manufactured (LMD) 316L microstructure in comparison to conventionally cast, as well as directionally solidified, 316L microstructures. The following conclusions were drawn:

- Local deformation behavior during scratching is highly influenced by the crystallographic orientation. Within individual grains, the scratch depth and pile-up behavior are relatively homogeneous, whereas localization of plastic deformation occurs at grain boundaries with high pile-ups.
- Scratching of individual grains with scratches smaller than the grain size, reveals that the scratch resistance is a function of grain orientation, and generally increases in the order (001)-(101)-(111). This tendency is independent of the investigated processing routes (conventional casting and LMD). The influence of the crystallographic orientation is also seen in the hardness measured by nanoindentation and correlates with the scratch depth.
- LMD processing leads to a reduction in scratch depth in comparison to casting. However, the deformation behavior in terms of pile-up height and the *f*_ab_ parameter are not significantly altered. Hence, LMD-processed microstructures show a higher local scratch resistance compared to the microstructures produced by conventional casting.
- The higher strength, hardness, and scratch resistance of LMD-processed microstructures is most likely due to a higher dislocation density. Pop-in phenomenon in the early beginning of the loading curve indicate a low defect density, and hence low dislocation density after the conventional casting route. The higher dislocation density and number of dislocation sources of the LMD-processed microstructure leads to early and continuous dislocation nucleation, and thus pop-ins are suppressed.
- A direct effect of the cellular substructure on the local deformation behavior during scratch and indentation testing is not seen. Pile-up behavior and occurring slip lines are not noticeably affected by the substructure.
- The indentation size effect (ISE) was investigated in nearly (001)-oriented grains. In the investigated grains, the ISE appeared to be of the same magnitude, independent of the processing route.

## Figures and Tables

**Table 1:** Parameters of the laser metal deposition (LMD) process.

Laser Power	Focus Diameter	Feed Rate	Powder Feed Rate	Hatch Distance	Layer Height
315 W	1 mm	10 mm/s	2.3 g/min	0.5 mm	0.3 mm

**Table 2:** Chemical composition of the investigated specimens measured by optical emission spectroscopy. Cast and LMD specimens were produced by the same gas atomized 316L powder. To avoid the evaporation of Mn, the composition was adjusted for directional solidification (DS). All values are given in mass%.

Processing Route	C	N	Mn	Si	Cr	Ni	Mo	S + P	Fe
Cast	0.07	0.04	0.73	0.82	16.67	12.71	2.47	0.02	bal.
LMD									
DS	0.03	0.04	0.08	0.02	17.65	11.08	2.43	0.01	bal.
